# Causal relationships of metabolites with allergic diseases: a trans-ethnic Mendelian randomization study

**DOI:** 10.1186/s12931-024-02720-6

**Published:** 2024-02-20

**Authors:** Junhao Tu, Jinyang Wen, Qing Luo, Xin Li, Deyun Wang, Jing Ye

**Affiliations:** 1 Department of Otorhinolaryngology, Head and Neck Surgery, The First Affiliated Hospital, Jiangxi Medical College, Nanchang University, Nanchang, Jiangxi Province China; 2 Department of Otolaryngology, Yong Loo Lin School of Medicine, National University of Singapore, National University Health System, Singapore, Singapore; 3 Department of Radiology, Tongji Hospital, Tongji Medical College, Huazhong University of Science and Technology, Wuhan, Hubei Province China; 4 Jiangxi Medicine Academy of Nutrition and Health Management, Nanchang, Jiangxi Province China; 5 Department of Allergy, The First Affiliated Hospital, Jiangxi Medical College, Nanchang University, Nanchang, Jiangxi Province China; 6 Institute of Otorhinolaryngology, The First Affiliated Hospital, Jiangxi Medical College, Nanchang University, Nanchang, Jiangxi Province China

**Keywords:** Allergic diseases, Allergic rhinitis, Asthma, Metabolites, Mendelian randomization

## Abstract

**Background:**

Allergic diseases exert a considerable impact on global health, thus necessitating investigations into their etiology and pathophysiology for devising effective prevention and treatment strategies. This study employs a Mendelian randomization (MR) analysis and meta-analysis to identify metabolite targets potentially associated with allergic diseases.

**Methods:**

A two-sample MR analysis was conducted to explore potential causal relationships between circulating and urinary metabolites and allergic diseases. Exposures were derived from a genome-wide association study (GWAS) of 486 circulating metabolites and a GWAS of 55 targeted urinary metabolites. Outcome data for allergic diseases, including atopic dermatitis (AD), allergic rhinitis (AR), and asthma, were obtained from the FinnGen biobank in Europe (cohort 1) and the Biobank Japan in Asia (cohort 2). MR results from both cohorts were combined using a meta-analysis.

**Results:**

MR analysis identified 50 circulating metabolites and 6 urinary metabolites in cohort 1 and 54 circulating metabolites and 2 urinary metabolites in cohort 2 as potentially causally related to allergic diseases. A meta-analysis of the MR results revealed stearoylcarnitine (OR 8.654; 95% CI 4.399−17.025; *P* = 4.06E-10) and 1-arachidonoylglycerophosphoinositol (OR 2.178; 95% CI 1.388−3.419; *P* = 7.15E-04) as the most reliable causal circulating metabolites for asthma and AR, respectively. Further, histidine (OR 0.734; 95% CI: 0.594−0.907; *P* = 0.004), tyrosine (OR 0.601; 95% CI: 0.380−0.952; *P* = 0.030), and alanine (OR 0.280; 95% CI: 0.125−0.628; *P* = 0.002) emerged as urinary metabolites with the greatest protective effects against asthma, AD, and AR, respectively.

**Conclusions:**

Imbalances in numerous circulating and urinary metabolites may be implicated in the development and progression of allergic diseases. These findings have significant implications for the development of targeted strategies for the prevention and treatment of allergic diseases.

**Supplementary Information:**

The online version contains supplementary material available at 10.1186/s12931-024-02720-6.

## Introduction

Allergic diseases affect a considerable percentage of the worldwide population [1]. These diseases, including allergic conjunctivitis (AC), atopic dermatitis (AD), allergic rhinitis/pollinosis (AR), and asthma, may progress in a stepwise manner [2, 3]. Recent epidemiological studies have shown an increasing trend in the prevalence of allergic diseases, with approximately 22% of residents in 30 countries affected [4]. Global public health is faced with a significant challenge as the severity and complexity of allergic diseases continue to rise [5]. Therefore, exploring the causal effects of allergic diseases is crucial in reducing the incidence of allergic diseases.

Numerous metabolites may play roles in the progression of allergic diseases. For instance, a correlation between the metabolites involved in linoleic acid and arachidonic acid metabolism and asthma control has been observed [6]. Additionally, Zheng et al. reported that the serum hydroxyeicosatetraenoic acid level was significantly decreased after subcutaneous immunotherapy in AR [7]. Further, various differential metabolites have been found in other types of allergic diseases, such as AC and AD [8–11]. Although some specific metabolites might influence the risk of allergic diseases and serve as strong indicators of an intervention effect, deciphering their roles in the development and progression of allergic diseases has been challenging. Randomized controlled trials are not feasible without sufficient meaningful evidence, and traditional observational studies may generate biases due to confounding factors. Importantly, genetic differences between Asian and European populations may affect the metabolism and development of allergic diseases. Therefore, conducting joint analyses in both Asian and European cohorts can help to better understand the roles of specific metabolites in the development and progression of allergic diseases. For example, differences in the metabolism of sphingolipids and amino acids between Asian and European populations have been reported [12]. Moreover, genetic variations in metabolism-related genes have been linked to the susceptibility and progression of allergic diseases [13, 14]. Thus, conducting analyses in both Asian and European cohorts can help to identify potential genetic and metabolic factors that contribute to the development and progression of allergic diseases.

Mendelian randomization (MR) is an innovative epidemiological approach that employs common genetic variants to serve as proxies for exposures, thus enabling the prediction of their causal association with the outcome [15–17]. As MR analysis is nearly impervious to confounding factors, it utilizes single nucleotide polymorphisms (SNPs) as instrumental variables (IVs). Therefore, MR analysis has been extensively utilized to evaluate the causal relationships between metabolite levels and various complex diseases, such as lung cancer [18], hypertension [19], non-alcoholic fatty liver [20], anxiety disorders [21], and COVID-19 [22].

In this MR study, our aim is to identify specific circulating and urinary metabolites with potential causal relationships with allergic diseases. We used 2 large-scale metabolomics datasets that included circulating non-targeted metabolomics and urinary targeted metabolomics, as well as the genome-wide association studies (GWAS) for 3 allergic diseases, namely, AD, AR, and asthma. By combining the results from the European database and Asian database, we investigated the circulating and urinary metabolites that are associated with the pathophysiology of allergic diseases. The results of this study will contribute to the identification of potential new therapeutic targets for allergic diseases.

## Materials and methods

### Mendelian randomization assumptions and study design

**Fig. 1:**
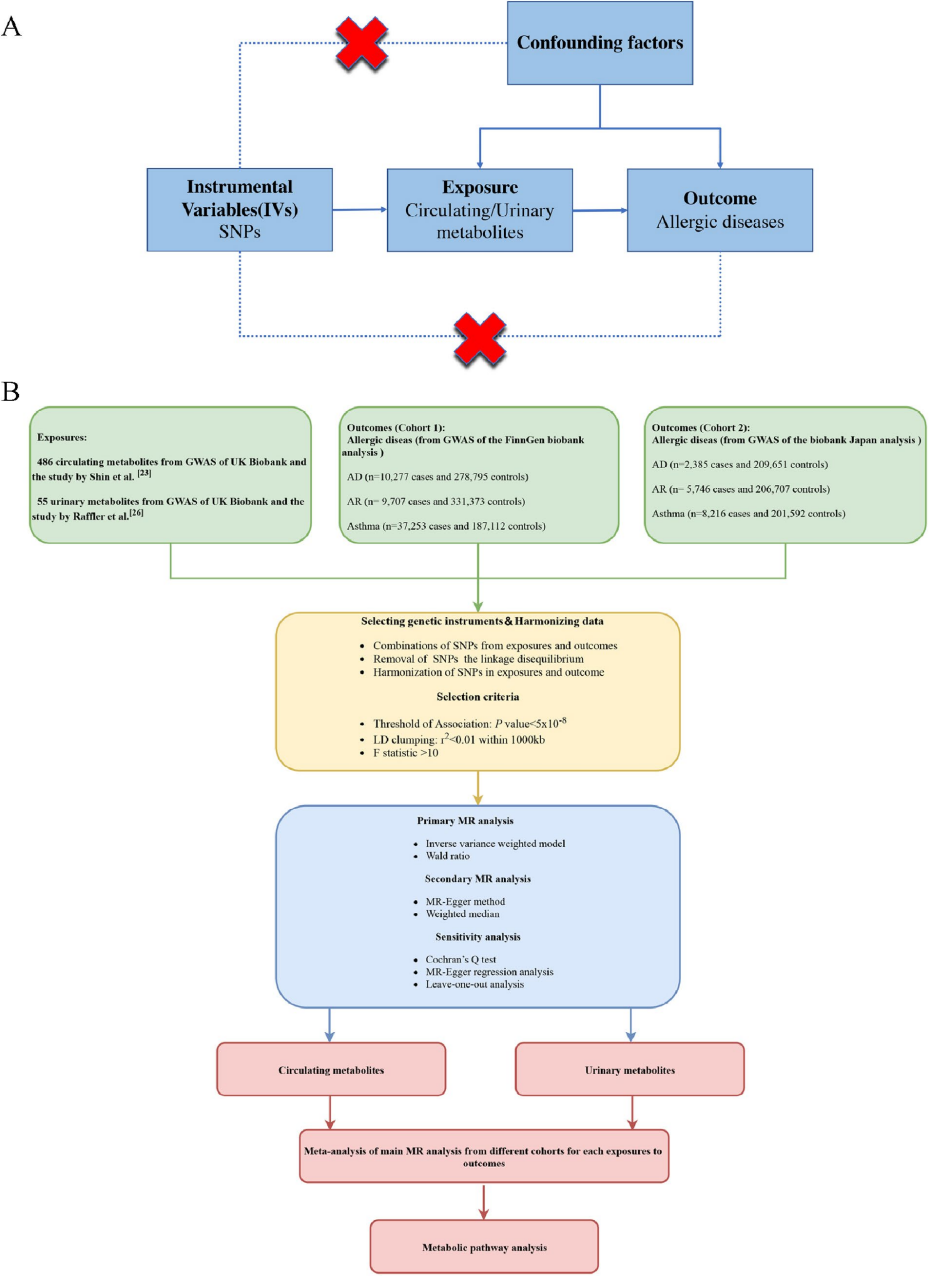
(**A**) Mendelian randomization key assumptions diagram. (**B**) Schematic design and overview flowchart of the hypothesis

MR is a method that utilizes genetic variants as IVs to infer causal relationships. Here, we used SNPs as proxies for exposures, abiding by the following 3 key assumptions that are essential for the validity of the SNPs as effective IVs (Fig. 1A). (1) Relevance: The genetic variant must be closely associated with the exposure, thus ensuring that the variant is a suitable proxy for the exposure in question. (2) Exclusion restriction: The genetic variant is related to the outcome only through the exposure, and no other pathways influence the outcome. (3) Independence: The association between the genetic variant and the outcome is not confounded by any potential confounding factors.

The workflow of this study is shown in Fig. 1B. We employed a two-sample MR to find the causal relationship between genetically determined circulating and urinary metabolites and allergic diseases. This is the first MR study to analyze the impact of circulating and urinary metabolites on allergic diseases from a genetic perspective. In addition, we conducted a metabolic pathway enrichment analysis to identify the pathways through which circulating and urinary metabolites may influence allergic diseases.

### Data source and genetic instrumental variables of the metabolites

The GWAS summary datasets for 486 circulating metabolites were obtained from Shin et al.’s study [23]. This study is currently the largest GWAS study on the genetic influences on human circulating metabolites; a total of 7,824 participants were recruited from 2 cohorts comprised of 1,768 participants from KORA F4 in Germany and 6,056 participants from the Twins UK cohort. The participants provided fasting serum samples that were analyzed following signed informed consent. Both of the studies were conducted in accordance with relevant ethical regulations and approved by the corresponding ethics committees. Genotyping information of both the KORA dataset and the Twins UK dataset has been described in previous studies [23–25]. The metabolites were analyzed using liquid-phase chromatography and gas chromatography (Metabolon, Inc.). In total, 486 circulating metabolites were identified and genetically analyzed, including 309 known and 177 unknown metabolites.

The GWAS summary dataset for urinary metabolites used in this study was obtained from Raffler et al. [26]. The dataset was based on 3,861 participants in the SHIP-0 cohort and 1,691 participants in the KORA F4 cohort. Metabolites were assayed using targeted metabolomics, and a total of 55 urinary targeted metabolites were included in our analysis.

Statistical associations between the metabolites and SNPs were summarized with genome-wide significance (*P* < 5.00E-8). The linkage disequilibrium (LD) was tested under the condition of the clumping procedure with r^2^ = 0.01 and a window size = 1000 kb. F statistics were used to minimize potential weak instrumental bias. The F statistic needs to meet at least 10 for performing an MR analysis [27, 28]. The exposures and outcomes were harmonized according to effect alleles, and subsequent analyses were conducted by the merged exposure-outcome dataset.

### GWAS data of allergic diseases

To evaluate the causal relationship between metabolites and allergic diseases, European genetic data of AD, AR, and asthma were obtained from the FinnGen biobank analysis (round 8), and diagnoses were based on International Classification of Diseases (ICD-10) (Fig. 1). The European cohort (cohort 1) was comprised of 3,846 cases and 306,909 controls for AD, 9,707 cases and 331,173 controls for AR, and 37,253 cases and 187,112 controls for asthma. We also obtained Asian genetic data from the Biobank Japan in order to find well-powered evidence for causal effects on the allergic disease risk. The Asian cohort (cohort 2) comprised 2,385 cases and 209,651 controls for AD, 5,746 cases and 206,707 controls for AR, and 8,216 cases and 201,592 controls for asthma.

### Two-sample Mendelian randomization

The MR analyses were performed using 4 different methods: the inverse variance weighted model (IVW), the wald ratio, the MR-Egger method, and the weighted median. The IVW model is a weighted linear regression model that aggregates multiple IVs, with each variable weighted in inverse proportion to its variance to minimize the overall variance. The IVW model calculates the causal estimates of each SNP and combines them using a meta-analysis to obtain a total causal estimate [29, 30]. The MR-Egger regression method was used to detect and correct for horizontal pleiotropy. The intercept of MR-Egger can be used as an indicator of unbalanced directional pleiotropy [27]. The weighted median method is a complementary method to the MR-Egger regression; it sorts MR estimates according to their inverse variances, and the weighted median estimate is the 50% weighted percentile. The weighted median can be considered an unbiased estimate of the causal effect in MR If more than 50% of the weight comes from effective SNPs [31]. In addition, the Wald ratio method was used to estimate the causal effects of individual SNPs; this method provides a ratio of the estimates of the SNP effect on the exposure and the outcome to estimate the causal effect [32].

For sensitivity analysis, we conducted a Cochran’s Q test, an MR-Egger regression analysis, and a leave-one-out analysis. Cochran’s Q test is used to explore the heterogeneity, and a *P* value less than 0.05 for Cochran’s Q test was considered as statistically significant heterogeneity [33]. A leave-one-out sensitivity analysis was used to monitor if significant associations were dominated by a single SNP.

### Pathway analysis of the circulating and urinary metabolites

Pathway analysis of the circulating metabolites and urinary metabolites was performed using the web-based tool MetaboAnalyst 5.0 (https://www.metaboanalyst.ca/MetaboAnalyst). Only metabolites that met the statistical significance threshold of *P* < 0.05 based on the full meta-analysis were included in the pathway analysis. Functional enrichment analysis and pathway analysis were conducted to identify potential metabolic pathways that may be associated with allergic diseases.

### Statistical analysis

To minimize the likelihood of false-positive results, the Bonferroni correction for multiple tests was performed. All of the *P* values were two-sided, *P* < 0.05/255 = 2.00E-4 for circulating metabolites and *P* < 0.05/18 = 2.80E-3 for urinary metabolites were considered to be statistically significant, and *P* < 0.05 but that which did not reach statistical significance was considered suggestively significant. All of the MR analyses were performed using R software (version 4.1.2) through the “TwoSampleMR” package and the “Mendelian Randomization” package [34]. To combine estimates from the GWAS studies, we employed a Cochran’s Q-statistics test to calculate both the I^2^ statistics and corresponding *P* value to assess the heterogeneity between estimates from the different databases. In addition, we conducted a full meta-analysis of all of the metabolite results to capture the broader associations within the data, followed by a subgroup meta-analysis of metabolites exhibiting potential causal associations (*P* < 0.05) with allergic diseases in both cohorts. We utilized the fixed-effect model meta-analyses to combine the estimates from different GWAS studies if no significant heterogeneity was detected across the databases for a specific exposure. However, in the presence of significant heterogeneity, we excluded any obvious clinical heterogeneity and then used the random-effect model meta-analyses to combine the estimates.

## Results

### Selection of the instrumental variables

Using the GWAS summary dataset of circulating metabolites, we identified a total of 9,472 SNPs associated with 260 metabolites based on the threshold of *P* < 5.00E-8. We retained 437 independent IVs from 255 circulating metabolites for further MR analysis after excluding IVs that were in linkage disequilibrium (LD) (r^2^ > 0.01) and in proximity (within 1000 kb), and harmonizing the SNPs in the exposures and outcomes (Table S1). For urinary metabolites, we applied the same screening criteria and obtained 26 independent SNPs associated with 18 metabolites for the MR analysis (Table S2).

### Causality of the genetically determined circulating metabolites on asthma

We analyzed 255 circulating metabolites for their associations with asthma in both cohorts. After that, we performed a full meta-analysis on the MR data in both cohorts.

In cohort 1, we found that 4 metabolites were significantly associated with asthma risk (*P* < 2.00E-4, Table 1): stearoylcarnitine (OR 7.151; 95% CI 3.392−15.074; *P* = 2.33E-07), 2-tetradecenoyl carnitine (OR 4.879; 95% CI 2.622−9.079; *P* = 5.68E-07), oleoylcarnitine (OR 5.969; 95% CI 2.964−12.020; *P* = 5.68E-07), and palmitoylcarnitine (OR 7.155; 95% CI 3.309−15.469; *P* = 5.68E-07). We also observed suggestive evidence of association for 20 other circulating metabolites (2.00E-4 < *P* < 0.05; Table S3), including X-11,483 (OR 0.53; 95% CI 0.376−0.747; *P* = 2.83E-04), 1-eicosadienoylglycerophosphocholine (OR 0.27; 95% CI 0.122−0.599; *P* = 1.27E-03), and adrenate (22:4n6) (OR 2.11; 95% CI 1.324−3.363; *P* = 1.68E-03).

**Table 1:** MR results in circulating metabolites from cohort 1

Outcome	Exposure	Method	SNPs	OR	95% CI	*P* value
Asthma	Stearoylcarnitine	Wald ratio	1	7.151	3.392–15.074	2.33E-07
	2-Tetradecenoyl carnitine	Wald ratio	1	4.879	2.622–9.079	5.68E-07
	Oleoylcarnitine	Wald ratio	1	5.969	2.964–12.020	5.68E-07
	Palmitoylcarnitine	Wald ratio	1	7.155	3.309–15.469	5.68E-07

(*P* < 0.0002)

OR: Odds Ratio, CIs: confidence intervals

We also observed similar results in cohort 2 as those in cohort 1. 2 circulating metabolites were significantly associated with asthma risk based on the genetic analysis (Table 2): oleoylcarnitine (OR 16.871; 95% CI 3.826−74.407; *P* = 1.90E-04) and palmitoylcarnitine (OR 23.024; 95% CI 4.434−119.559; *P* = 1.90E-04). Additionally, we found that acetylcarnitine (OR 0.023; 95% CI 0.003−0.163; *P* = 1.90E-04) significantly exerted a protective effect in asthma. However, 2-tetradecenoyl carnitine (OR 0.076; 95% CI 0.020−0.289; *P* = 1.54E-04) showed the opposite results in cohort 2 compared to cohort 1. We also observed suggestive evidence of association for 37 other circulating metabolites (Table S4).

**Table 2:** MR results in circulating metabolites from cohort 2

Outcome	Exposure	Method	SNPs	OR	95% CI	*P* value
Asthma	2-Tetradecenoyl carnitine	Wald ratio	1	0.076	0.020–0.289	1.54E-04
	Acetylcarnitine	Wald ratio	1	0.023	0.003–0.163	1.54E-04
	Oleoylcarnitine	Wald ratio	1	16.871	3.826–74.407	1.90E-04
	Palmitoylcarnitine	Wald ratio	1	23.024	4.434–119.559	1.90E-04

(*P* < 0.0002)

OR: Odds Ratio, CIs: confidence intervals

**Fig. 2:**
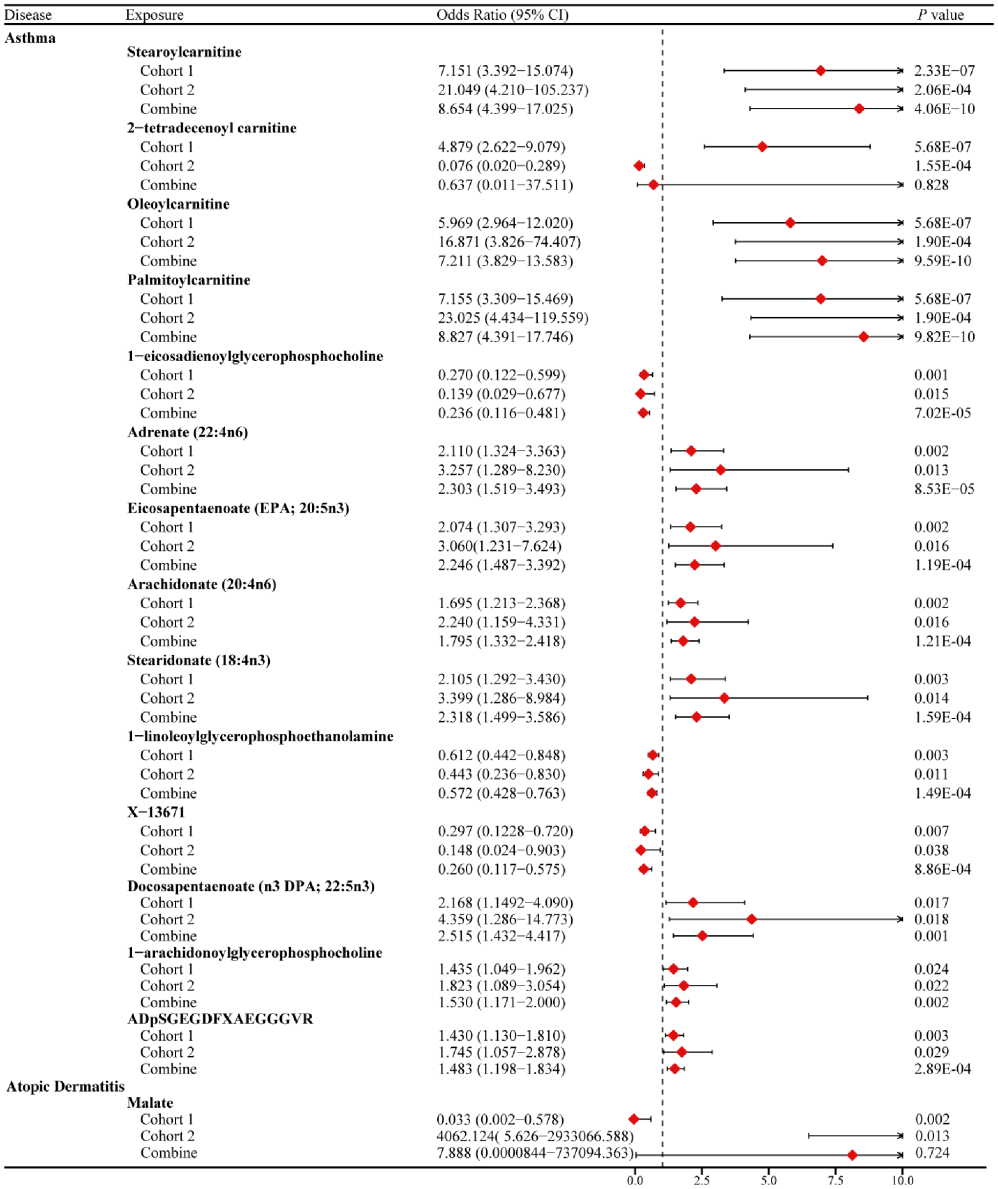
This forest plot displays the results of the subgroup meta-analyses conducted on shared circulating metabolites indicative of allergic diseases associations in both cohorts (*P* value < 0.05 in both cohorts), examining their potential links to the allergic disease risk. CIs: confidence intervals

Further, we conducted a full meta-analysis of the circulating metabolite results from both cohorts. We found that 16 circulating metabolites were identified as risk factors for asthma, while 8 circulating metabolites exhibited a protective effect against asthma (Figure S1 and Table S5). We also found that 14 circulating metabolites consistently showed association with asthma in both cohorts based on the MR analyses (*P* < 0.05 at both cohorts). A subgroup meta-analysis of these 14 circulating metabolites revealed that high genetic serum expression levels of stearoylcarnitine (OR 8.654; 95% CI 4.399−17.025; *P* = 4.06E-10), oleoylcarnitine (OR 7.211; 95% CI 3.829−13.583; *P* = 9.59E-10), palmitoylcarnitine (OR 8.827; 95% CI 4.391−17.746; *P* = 9.82E-10), adrenate (22:4n6) (OR 2.303; 95% CI 1.519–3.493; *P* = 8.53E-05), eicosapentaenoate (EPA;20:5n3) (OR 2.246; 95% CI 1.487−3.392; *P* = 1.19E-04), arachidonate (20:4n6) (OR 1.795; 95% CI 1.332−2.418; *P* = 1.21E-04), stearidonate (18:4n3) (OR 2.318; 95% CI 1.499−3.586; *P* = 1.59E-04), docosapentaenoate (n3 DPA;22:5n3) (OR 2.515; 95% CI 1.432−4.417; *P* = 0.001), 1-arachidonoylglycerophosphocholine (OR 1.530; 95% CI 1.171−2.000; *P* = 0.002), and ADPSGEGDFXAEGGGVR (OR 1.483; 95% CI 1.198−1.834; *P* = 2.89E-04) were associated with an increased risk of asthma. In contrast, 1-eicosadienoylglycerophosphocholine (OR 0.236; 95% CI 0.116−0.481; *P* = 7.02E-05), 1-linoleoylglycerophosphoethanolamine (OR 0.572; 95% CI 0.428−0.763; *P* = 1.49E-04), and X-13,671 (OR 0.260; 95% CI 0.117−0.575; *P* = 8.86E-04) were found to have a protective effect against the occurrence of asthma (Fig. 2).

### Causality of the genetically determined circulating metabolites on atopic dermatitis

No significant associations were detected between the circulating metabolites and AD, but we did find suggestive evidence of association between 18 circulating metabolites and the risk of AD in cohort 1 (Table S6). For example, genetically high levels of X-11,478 showed suggestive evidence of association with AD (OR 2.73; 95% CI 1.323–5.637; *P* = 0.006). Additionally, we found potential inverse associations between genetically determined levels of X-11,444 (OR 0.12; 95% CI 0.369–0.4; *P* = 5.21E-04) and scyllo-inositol (OR 0.19; 95% CI 0.058–0.599; *P* = 0.005) in our study.

Additionally, there was suggestive evidence of an association between 8 circulating metabolites and the risk of AD in cohort 2 (Table S7). After conducting a full meta-analysis of data from both cohorts, we identified a potential inverse association between 16 circulating metabolites and AD that is determined by genetic factors. For example, 5-oxoproline (OR 0.099; 95% CI 0.027–0.364; *P* = 5.02E-4) and eicosapentaenoate (EPA; 20:5n3) (OR 0.239; 95% CI 0.083–0.691; *P* = 0.008) showed a potential inverse association. In contrast, there were 9 circulating metabolites with a high genetic correlation with AD that showed a potential positive causal relationship with the disease. For instance, X-11,452 (OR 4.811; 95% CI 1.583–14.616; *P* = 0.006) and 1-linoleoylglycerophosphoethanolamine (OR 2.512; 95% CI 1.197–5.271; *P* = 0.015) (Figure S2, Table S8). We found that malate showed evidence of association with AD in both the cohorts based on the MR analyses. However, the subgroup meta-analysis revealed no evidence of association between malate and AD (OR: 7.888; 95% CI: 0.0000844−737094.363; *P* = 0.724) (Fig. 2).

### Causality of genetically determined circulating metabolites on allergic rhinitis

No significant associations were detected between circulating metabolites and AR in both cohorts; however, in cohort 1, 21 circulating metabolites showed suggestive evidence of association with the risk of AR (Table S9). For example, genetically high level of 1-arachidonoylglycerophosphoinositol showed suggestive evidence for association with AR (OR 2.625; 95% CI 1.486–4.64; *P* = 8.93E-04). In addition, we found potential inverse associations between genetically determined levels of 1-eicosadienoylglycerophosphocholine (OR 0.117; 95% CI 0.028–0.494; *P* = 3.51E-03) and scyllo-inositol (OR 0.327; 95% CI 0.154–0.695; *P* = 3.70E-03) in our study.

We identified suggestive evidence for an association between 11 circulating metabolites and AR in cohort 2 (Table S10). To investigate further, we performed a full meta-analysis of both cohorts that revealed 9 circulating metabolites that may serve as protective factors for the development of AR, including kynurenine (OR 0.363; 95% CI 0.193–0.684; *P* = 0.002). Another potential protective factor was 1-eicosadienoylglycerophosphocholine (OR 0.182; 95% CI 0.057–0.578; *P* = 0.004). Conversely, we also found that 13 circulating metabolites may be potential risk factors for AR, such as 1-arachidonoylglycerophosphoinositol (OR 2.178; 95% CI 1.388–3.419; *P* = 7.15E-04) and stearidonate (18:4n3) (OR 2.661; 95% CI 1.309–5.410; *P* = 0.007) (Figure S3, Table S11).

### Causality of the genetically determined urinary metabolites on allergic diseases

There were no significant associations of the urinary metabolites with any allergic diseases in both cohorts (*P* < 0.0028), but there are some potential causal effects were detected in cohort 1 (Table S12). Specifically, lysine (OR = 1.272; 95% CI: 1.003−1.612; *P* = 0.047) and glycolate (OR = 1.852; 95% CI: 1.082−3.170; *P* = 0.025) were the most dangerous urinary metabolites with high risk to cause AR, and asthma, respectively (Table 3). Furthermore, results showed that histidine had a protective value against AR and asthma. Histidine could lower the incidence of AR (OR = 0.555; 95% CI: 0.359−0.856; *P* = 0.008) and asthma (OR = 0.759; 95% CI: 0.597−0.964; *P* = 0.024) (Table 3).

**Table 3:** MR results in urinary metabolites from cohort 1

Outcome	Exposure	Method	SNPs	OR	95% CI	*P* value	Heterogeneity test Q (*P* value)	MR-Egger pleiotropy test Intercept (*P* value)
Allergic Rhinitis	Histidine	IVW	2	0.555	0.359−0.856	0.008	0.399	NA
	Alanine	Wald ratio	1	0.307	0.108−0.875	0.027	NA	NA
	Threonine	Wald ratio	1	0.2634	0.081−0.861	0.027	NA	NA
	Lysine	Wald ratio	1	1.272	1.003−1.612	0.047	NA	NA
	Maleate	Wald ratio	1	1.400	1.004−1.951	0.047	NA	NA
Asthma	Histidine	IVW	2	0.759	0.597−0.964	0.024	0.767	NA
	Glycolate	Wald ratio	1	1.852	1.082−3.170	0.025	NA	NA
	Tyrosine	IVW	3	0.742	0.567−0.972	0.030	0.559	0.686
	O.Phosphocholine	Wald ratio	1	0.516	0.281−0.947	0.033	NA	NA

(*P* < 0.05)

OR: Odds Ratio, CIs: confidence intervals

In cohort 2, interestingly, both threonine (OR 0.203; 95% CI: 0.048−0.857; *P* = 0.030) and alanine (OR 0.244; 95% CI: 0.068−0.872; *P* = 0.030) were found to be urine metabolites with potential protective value against both AD and AR (Table 4, Table S13).

**Table 4:** MR results in urinary metabolites from cohort 2

Outcome	Exposure	Method	SNPs	OR	95% CI	*P* Value
Atopic Dermatitis	Threonine	Wald ratio	1	0.203	0.048−0.857	0.030
	Alanine	Wald ratio	1	0.244	0.068−0.872	0.030
Allergic Rhinitis	Threonine	Wald ratio	1	0.203	0.048−0.857	0.030
	Alanine	Wald ratio	1	0.244	0.068−0.872	0.030

(*P* < 0.05)

OR: Odds Ratio, CIs: confidence intervals

**Fig. 3:**
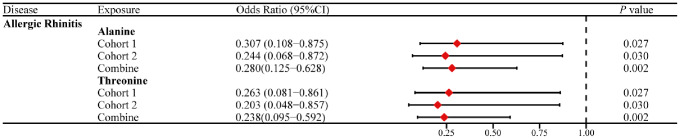
This forest plot displays the results of the subgroup meta-analyses conducted on shared urinary metabolites indicative of allergic diseases associations in both cohorts (*P* value < 0.05 in both cohorts), examining their potential links to the allergic disease risk. CIs: confidence intervals

After conducting a full meta-analysis of data from the 2 cohorts, we identified 7 urinary metabolites associated with allergic diseases. For example, histidine (OR 0.734; 95% CI: 0.594−0.907; *P* = 0.004), tyrosine (OR 0.601; 95% CI: 0.380−0.952; *P* = 0.030), and alanine (OR 0.280; 95% CI: 0.125−0.628; *P* = 0.002) were identified as factors with the highest protective value urinary metabolites for asthma, AD, and AR, respectively (Figure S4 A-C and Table S14). We observed that high levels of alanine and threonine in urine were consistently identified as protective factors against AR in both cohorts. A subgroup meta-analysis of the MR results further confirmed that high levels of alanine (OR 0.280; 95% CI: 0.125−0.628; *P* = 0.002) and threonine (OR 0.238; 95% CI:0.095−0.592; *P* = 0.002) in urine were associated with a decreased risk of AR (Fig. 3).

### Circulating and urinary metabolic pathway analysis

**Fig. 4:**
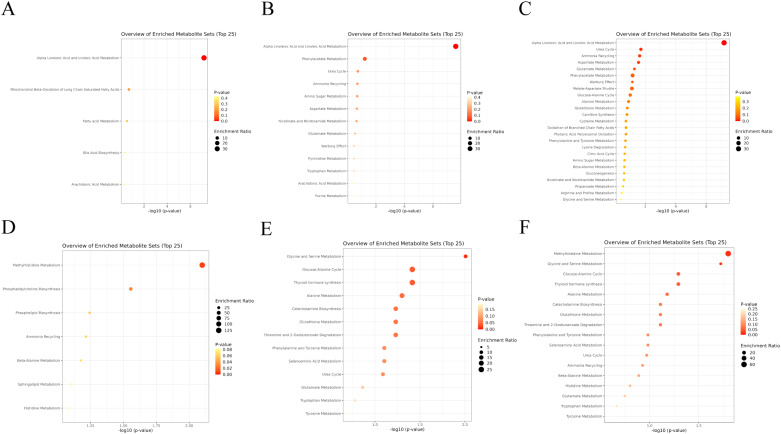
Bubble plot of the pathway enrichment analysis for the circulating and urinary metabolites based on a full meta-analysis of the MR analysis results. **(A-C)**. Bubble plot of the pathway enrichment analysis for the circulating metabolites in asthma **(A)**, AD **(B)**, AR **(C)**. **(D-F)**. Bubble plot of the pathway enrichment analysis for the urinary metabolites in asthma **(D)**, AD **(E)**, and AR **(F)**

To explore the potentially significant biological processes underlying allergic diseases, we conducted functional enrichment and pathway analyses using the metabolite results from the full meta-analysis. Our findings revealed that in circulation, the “Alpha Linolenic Acid and Linoleic Acid Metabolism”, “Urea Cycle”, and “Ammonia Recycling”, and “Aspartate Metabolism” pathway may be associated with AD, with *P*-values of 6.77E-08, 0.020, 0.024, and 0.028, respectively. Furthermore, the “Alpha Linolenic Acid and Linoleic Acid Metabolism” pathways may be linked to AR (*P* = 2.57E-08) and asthma (*P* = 6.77E-08) (Fig. 4A-C, Table S15). Additionally, our research has identified certain metabolic pathways in urine that may be associated with allergic diseases. For instance, pathways such as “Glycine and Serine Metabolism”, “Glucose-Alanine Cycle”, and “Thyroid hormone synthesis” have been found to correlate with AD, with *P*-values of 0.010, 0.039, and 0.039, respectively. Moreover, “Methylhistidine Metabolism” and “Glycine and Serine Metabolism” are associated with AR, with *P*-values of 0.016 and 0.019, respectively. “Methylhistidine Metabolism” (*P* = 7.97E-03) and “Phosphatidylcholine Biosynthesis” (*P* = 0.028) have been shown to be relevant with asthma (Fig. 4D-F, Table S16).

## Discussion

**Fig. 5:**
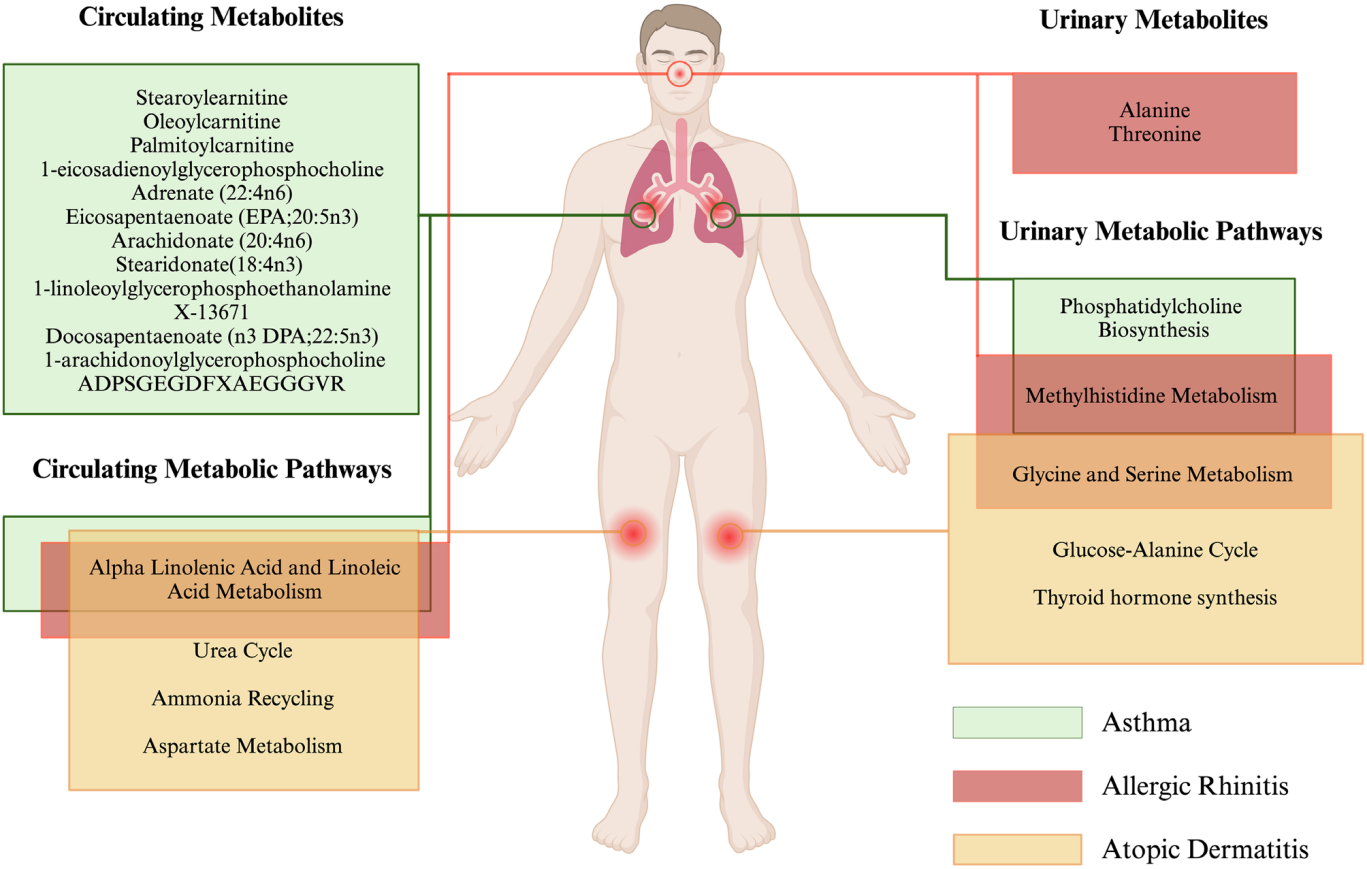
This illustration highlights the causal metabolites and their interconnected metabolic pathways related to allergic diseases

In this study, we conducted MR analyses in 2 cohorts to evaluate the causal relationship between circulating metabolites and urinary metabolites with allergic diseases, namely, AD, AR, and asthma. We identified 50 circulating metabolites and 6 urinary metabolites relevant to the risk of three allergic diseases in cohort 1 using genetic variants as probes. After the Bonferroni correction, stearoylcarnitine, 2-tetradecenoyl carnitine, oleoylcarnitine, and palmitoylcarnitine were identified as strong causal factors for the risk of asthma. In cohort 2, we identified 54 circulating metabolites and 2 urinary metabolites associated with the risk of 3 allergic diseases using genetic variants as probes. Moreover, a full meta-analysis of the data from the 2 cohorts revealed that 22 circulating metabolites were associated with the occurrence of AR, 25 circulating metabolites were associated with AD, and 24 circulating metabolites were associated with asthma. Additionally, we identified 7 urinary metabolites related to the pathogenesis of allergic diseases. Finally, the results of pathway analysis based on the meta-analysis showed that 4 circulating metabolic pathways and 5 urinary metabolic pathways may be involved in the biological processes of 3 allergic diseases (Fig. 5).

We identified 2 circulating metabolites that were significantly associated with the genetic risk of asthma in both cohorts, as well as in the subgroup meta-analysis. While 2-tetradecenoyl carnitine showed an association with asthma in both cohorts, the causal effects were markedly different, thus reflecting the heterogeneity between the two races; therefore, performing a meta-analysis of the MR results was necessary. The subgroup meta-analysis revealed a strong genetic association between circulating levels of stearoylcarnitine and asthma. A prior untargeted metabolomics analysis by Shi et al. from human immunodeficiency virus (HIV)-infected individuals found that high levels of stearoylcarnitine, oleoylcarnitine, and palmitoylcarnitine were related to poor immune recovery in HIV-infected patients. Moreover, the levels of stearoylcarnitine, oleoylcarnitine, and palmitoylcarnitine had negative correlations with the CD4^+^ T cell count in immunological non-responders [35]. Asthma is an inflammatory disease of the airways mediated by CD4^+^ T helper (Th) cells that include Th1, Th2, and Th17 cells. The interaction between cytokines and CD4^+^ T cells contributes to the progression of severe asthma [36]. For a long time, Th2 cells were considered a major cause of morbidity in patients with asthma, while Th1 cells were thought to play a protective role in asthma [37]. However, a recent study found that Th2 and Th17 cells are reciprocally regulated in asthma [37], thus suggesting that an imbalance in CD4^+^ T cells may lead to the development of progressive inflammatory and allergic diseases such as asthma. Studies have shown that palmitoylcarnitine can induce apoptosis in CD4^+^CD25^+^ T cells and Jurkat cells by promoting caspase-3/7 and caspase-8 [38, 39]. Additionally, palmitoylcarnitine can trigger an inflammatory response by activating the JNK/ERK pathway in C2C12 cells [40]. The activation of the JNK/ERK pathway can induce apoptosis in human bronchial epithelial cells [41]. Our findings confirmed these results of previous studies. We speculated that circulating palmitoylcarnitine may induce apoptosis of certain subtypes of CD4^+^ T cells and human bronchial epithelial cells, leading to the occurrence of asthma. Based on these findings, palmitoylcarnitine may be a promising therapeutic target in asthma, although the underlying mechanisms remain unclear and further experimental research is required. There is also some evidence showing that the level of circulating stearoylcarnitine is related to parasitic infections and inflammation, both of which are thought to play a role in the development and exacerbation of asthma [42, 43]. We found that circulating stearoylcarnitine levels were significantly associated with the risk of asthma, suggesting that it may be a potential biomarker for asthma. However, further research is needed to fully understand the relationship between stearoylcarnitine and asthma and whether it may be a useful target for therapeutic intervention.

In addition, several metabolites previously reported to be associated with allergic diseases were identified in this study. L-tryptophan can be catabolized by Indoleamine 2,3-dioxygenase (IDO) in the N-formyl kynurenine pathway to produce kynurenine. It has been shown that asthma patients have low IDO-1 activity levels and high levels of tryptophan in lung tissue [44]. Similarly, the activity level of IDO-1 in the nasal epithelial cells of AR patients and those exposed to house dust mites is decreased [45, 46]. Furthermore, an in vitro study demonstrated that high levels of serum tryptophan in patients with AR are associated with an ineffective immunotherapy response [47]. However, the tryptophan metabolite, kynurenine, produced by IDO, has been shown to have a protective effect against Th2-mediated allergic airway inflammation, and the metabolites of kynurenine can stimulate T cell apoptosis and the Th1/Th2 polarization response, thus affecting the progression of allergic diseases [48]. Given that allergic airway inflammation is a common feature in the development of systemic allergic diseases, kynurenine has the potential to be an important target for predicting such diseases. The results of this study are consistent with previous findings, indicating a potential association between kynurenine and AR, highlighting kynurenine as a valuable target for allergic diseases and potentially a useful target for predicting disease prognosis.

This study also yielded interesting findings regarding urinary metabolites and their association with allergic diseases. In both cohorts, we observed suggestive evidence that high levels of threonine and alanine in urine are protective against AR. Furthermore, after conducting a meta-analysis of the MR results from both cohorts, we found that histidine in urine has a protective effect against both AR and asthma, while high levels of alanine, threonine, and tyrosine in urine are protective against AR and AD. These findings suggest that these urinary metabolites may be closely related to the development of allergic diseases. A metabolomics study on AR patients found a significant decrease in serum levels of alanine during the AR attack period [49]. This result was consistent with another metabolomics study on systemic lupus erythematosus where alanine, as the major substrate for gluconeogenesis, inhibited the glycolysis pathway [50]. These findings suggest the upregulation of the glycolysis pathway in AR patients during the onset period. Previous studies have also found that the tyrosine metabolism pathway is correlated with the severity of symptoms in patients with AR due to house dust mite allergy [51]. Moreover, a study on the HLA gene locus variation in specific dermatitis found that the HLA-B residue of 80 (T-threonine) is related to the remission of AD [52]. These results support our findings that urinary alanine and threonine levels may have a protective effect on allergic diseases. Mast cell degranulation is the primary pathogenic mechanism of allergic respiratory diseases such as AR and asthma. After exposure to pathogens, mast cells often release key inflammatory mediators such as histamine and leukotrienes. Histamine is generated by the decarboxylation of histidine by histidine decarboxylase, and therefore histidine is often closely associated with allergic diseases [53, 54]. The results of our study also suggest that histidine may be an important target for allergic diseases, especially AR and asthma.

Our circulating metabolic pathway analysis identified the “Alpha Linolenic Acid and Linoleic Acid Metabolism” pathway as the most significantly associated with allergic diseases. Linoleic acid (LA, 18:2n-6) and alpha-linolenic acid (α-LNA, 18:3n-3) are essential fatty acids that cannot be synthesized de novo, and their levels in humans depend primarily on dietary intake and the activity of various fatty acid metabolic enzymes. Derivatives of these fatty acids can be classified as omega-6 polyunsaturated fatty acids (PUFAs), represented by arachidonic acid and omega-3 PUFAs, represented by eicosapentaenoic acid (EPA, 20:5n-3) and docosahexaenoic acid (DHA, 22:6n-3), respectively [55]. During the progression of allergic diseases, the levels of omega-6 PUFAs increase in the body. For example, arachidonic acid plays a crucial role in allergic diseases, as it generates leukotrienes and prostaglandins, such as prostaglandin E2, which can induce the production of Th2-type cytokines, such as IL-4, IL-5, and IL-13, as well as the synthesis of IgE. These inflammatory mediators can activate inflammatory cells, such as mast cells and eosinophils, leading to allergic inflammatory responses [56, 57]. In contrast, omega-3 PUFAs are believed to play a critical protective role against allergic diseases. It has been reported that alpha-linolenic acid can suppress allergic reactions by inhibiting IgE-mediated Ca2^+^ mobilization, degranulation, and cytokine release in mast cells [58]. There is substantial evidence indicating that omega-3 PUFAs possess anti-inflammatory properties. For instance, the pregnancy diet intake guidelines from the UK suggest that pregnant women consume omega-3 PUFAs as supplements to reduce the likelihood of eczema and allergic diseases in their offspring. Similarly, Australian guidelines state that the maternal intake of omega-3 PUFAs can reduce the incidence of eczema in their offspring [59]. A prospective study has also shown that the intake of omega-3 PUFAs during childhood or adolescence may be associated with a reduced risk of developing asthma [60]. Collectively, these findings suggest that the “Alpha Linolenic Acid and Linoleic Acid Metabolism” pathway may play a significant role in the pathophysiology of allergic diseases.

This study has some limitations. First, the limited data available restricted our analysis to a narrow range of allergic diseases; therefore, we were unable to investigate additional types of allergic diseases. Second, due to the lack of Asian ancestry in the exposure data, there was a discrepancy in the racial composition between the exposure and the outcomes; therefore, we cannot consider the results from cohort 2 as indicative of Asian-specific results. Third, although MR analysis is a potent tool for evaluating the causal relationship between human circulating metabolites and allergic diseases, it is necessary to validate the findings of this study with basic experimental data. Fourth, the size of the sample is crucial for ensuring the accuracy of the MR analysis in assessing the genetic influence on metabolic products; therefore, in future studies, it will be necessary to increase the sample size to obtain more precise analytical results. Fifth, due to the limited data, we used metabolites with uncorrected *P*-values for the meta-analysis. Finally, although this study identified numerous metabolic products linked to the risk of allergic diseases, further investigation is necessary to determine their roles in the pathogenesis of allergic diseases.

## Conclusion

In summary, this MR study identified 50 circulating metabolites and 6 urinary metabolites in cohort 1, and 54 circulating metabolites and 2 urinary metabolites in cohort 2 that may have causal relationships with the pathogenesis of allergic diseases. Furthermore, after combining the MR results from both ethnic groups using a meta-analysis, a total of 62 circulating metabolites and 7 urinary metabolites were found to be related to the development of allergic diseases. This study also identified 4 circulating metabolic pathways and 5 urinary metabolic pathways that may be associated with allergic diseases. Overall, the development and progression of allergic diseases could be linked to an imbalance of numerous circulating and urinary metabolites. These findings carry significant implications for the development of effective strategies for the prevention and treatment of allergic diseases.

### Electronic supplementary material

Below is the link to the electronic supplementary material.

Supplementary Material 1

Supplementary Material 2

## Data Availability

No datasets were generated or analysed during the current study.
