# Supplementary material for: Causal relationships of metabolites with allergic diseases: a trans-ethnic Mendelian randomization study
Source: Respir Res. 2024 Feb 20;25:94. doi: 10.1186/s12931-024-02720-6 (PMC10880354; doi:10.1186/s12931-024-02720-6)

Figure S1. This forest plot displays the results of a full meta-analyses conducted on circulating metabolites indicative of Asthma associations in both cohorts, examining their potential links to the allergic disease risk. CIs: conﬁdence intervals.


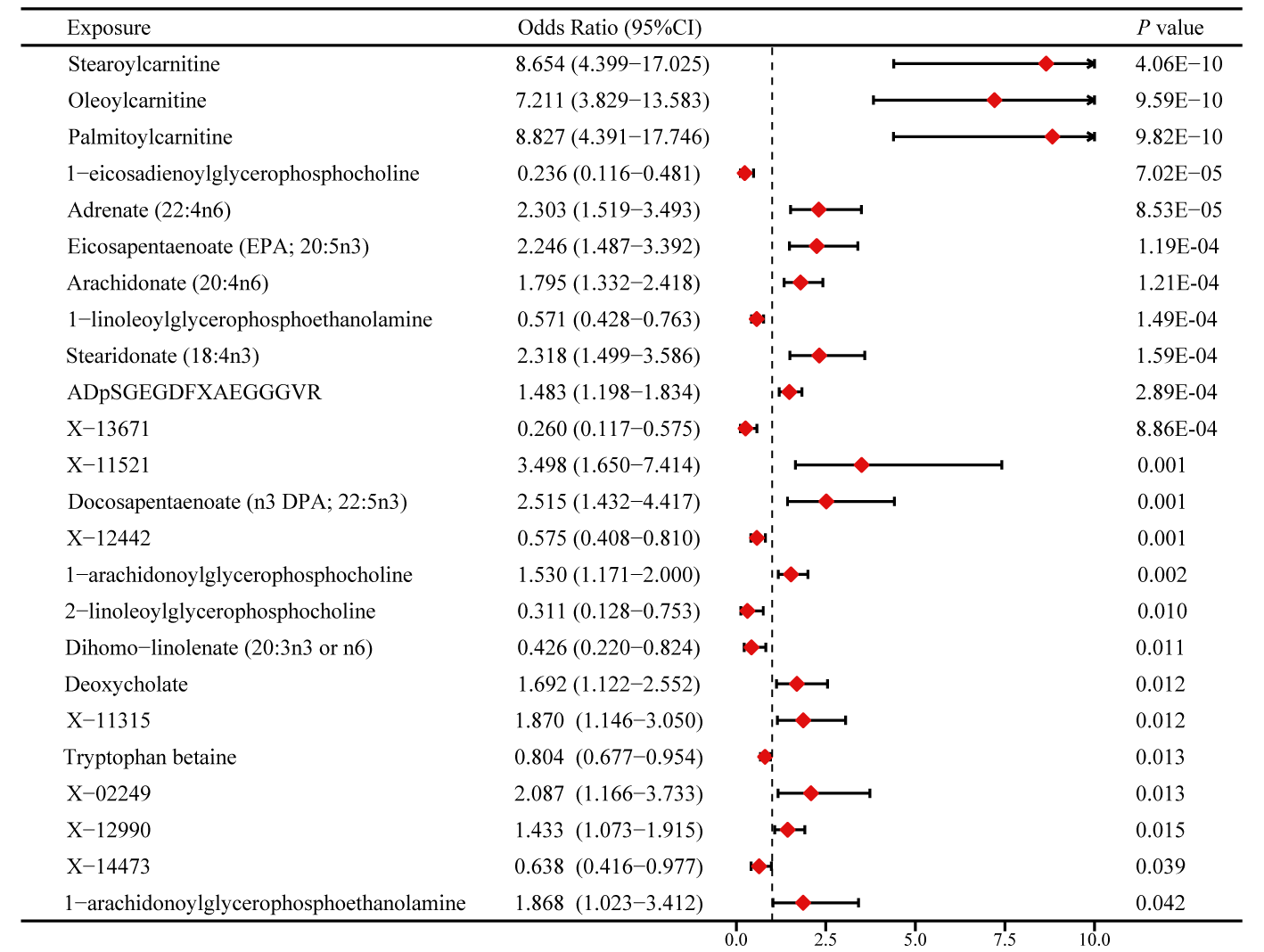


Figure S2. This forest plot displays the results of a full meta-analyses conducted on circulating metabolites indicative of AD associations in both cohorts, examining their potential links to the allergic disease risk. CIs: conﬁdence intervals.


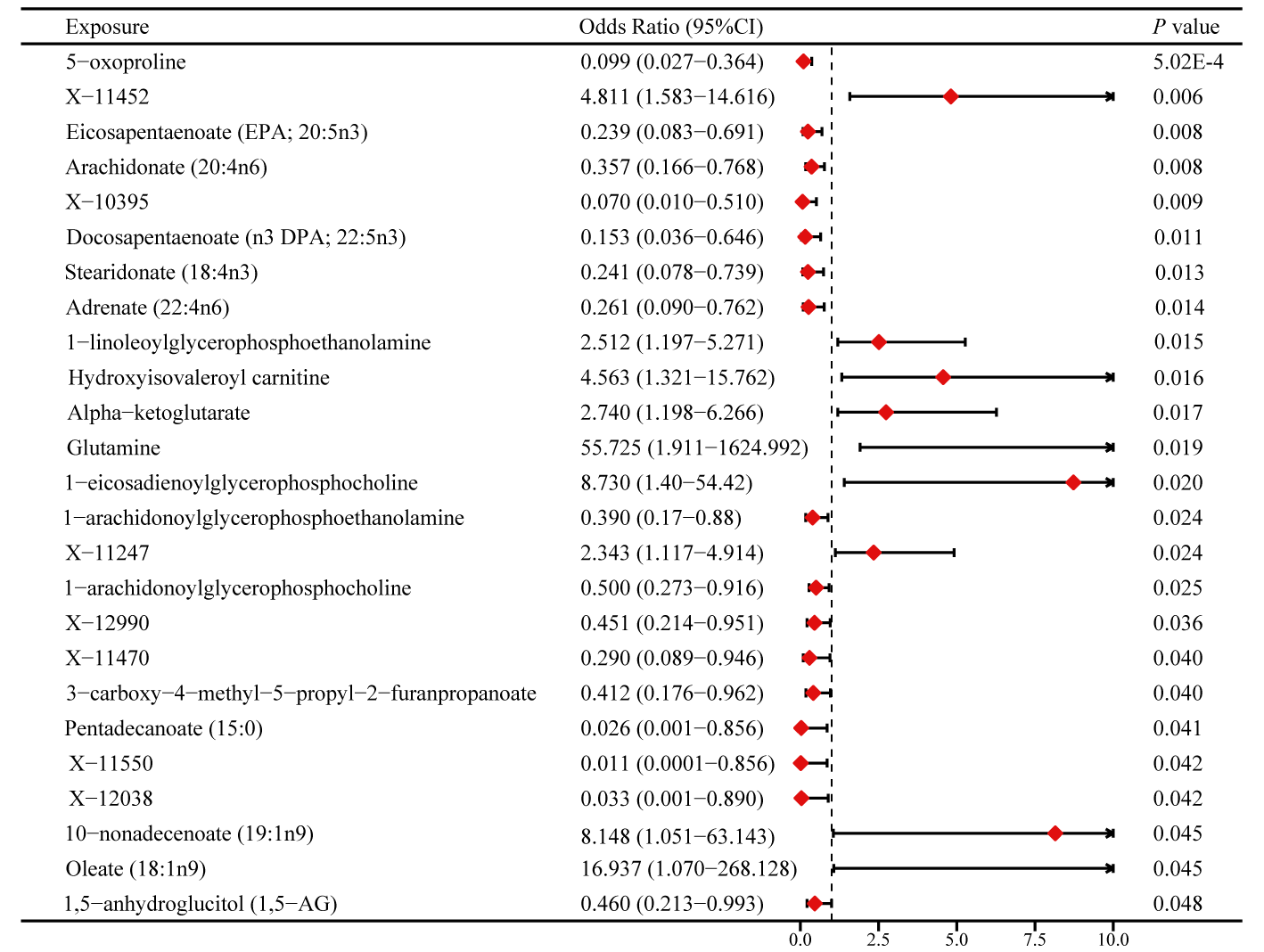


Figure S3. This forest plot displays the results of a full meta-analyses conducted on circulating metabolites indicative of AR associations in both cohorts, examining their potential links to the allergic disease risk. CIs. conﬁdence intervals.


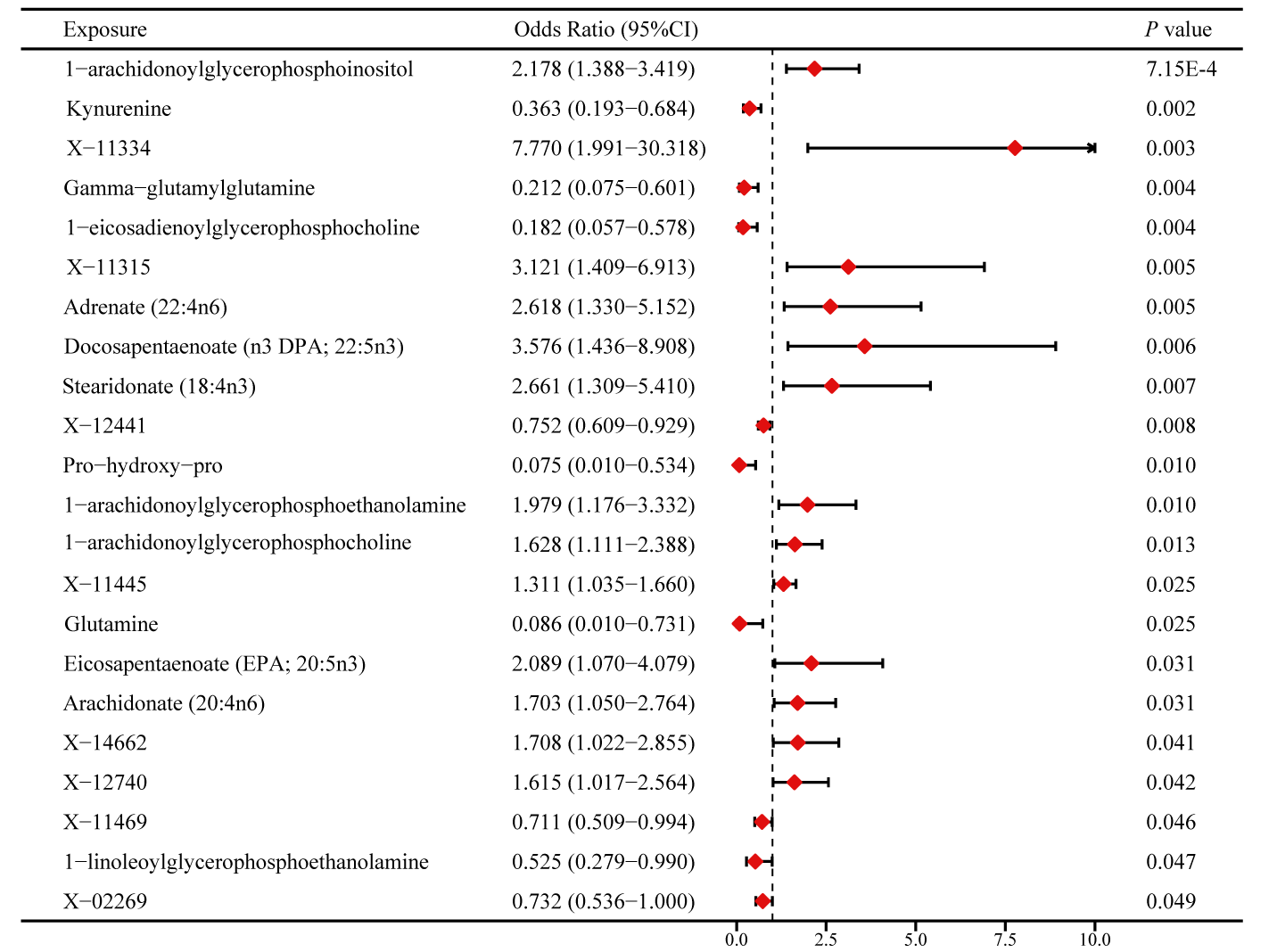


Figure S4. This forest plot displays the results of a full meta-analyses conducted on shared urinary metabolites indicative of asthma (**A**), AD (**B**), and AR (**C**) associations in both cohorts, examining their potential links to the allergic disease risk. CIs: conﬁdence intervals.


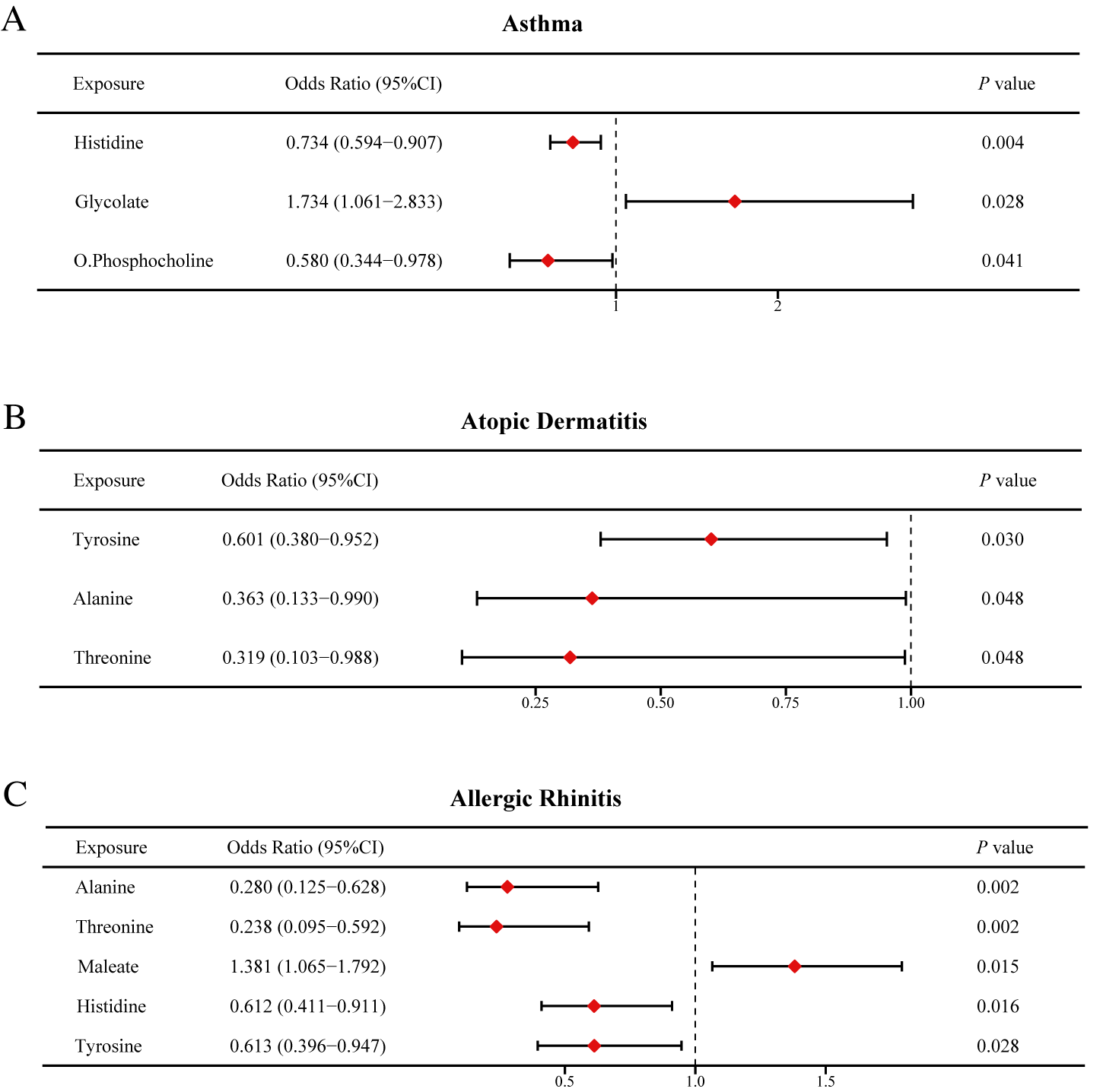

Supplement: Supplementary file 1 — Supplementary Material 1 [file 12931_2024_2720_MOESM1_ESM.docx]
